# Serum ferritin and the risk of myocardial infarction: A Mendelian randomization study

**DOI:** 10.1097/MD.0000000000037952

**Published:** 2024-04-26

**Authors:** Jianwei Zhou

**Affiliations:** a People’s Hospital of Xishuangbanna Dai Autonomous Prefecture, Jinghong, Yunnan, China.

**Keywords:** ferritin, Mendelian randomization, myocardial infarction

## Abstract

The potential role of serum ferritin as a risk factor for myocardial infarction (MI) is controversial, necessitating a systematic exploration of the causal relationship between ferritin and MI through Mendelian randomization (MR) methods. Genetic data were derived from a genome-wide association study (GWAS), employing the inverse variance-weighted (IVW) method as the primary approach. Comprehensive sensitivity analyses were conducted to validate the robustness of the results. Evaluation of instrumental variables was performed using the F-statistic, and a meta-analysis was employed to assess the average gene-predicted effect between ferritin and MI. The MR study revealed a negative correlation between ferritin and MI. The odds ratios (ORs) in the IVW method were 0.83 [95% confidence interval (CI) = 0.72–0.97; *P* = .017] and 0.86 (95% CI = 0.72–1.02; *P* = .080). Additionally, meta-analysis consistently indicated a negative causal relationship between ferritin and MI, with no heterogeneity or horizontal pleiotropy, thereby indicating a negative correlation between ferritin levels and the risk of MI. The genetic evidence sheds light on the causal relationship between ferritin levels and MI risk, providing new perspectives for future interventions in acute myocardial infarction (AMI).

## 1. Introduction

Iron, a crucial mineral element, plays a pivotal role in numerous fundamental biological processes, including facilitating catalytic reactions,^[1]^ sustaining cellular respiration,^[2]^ and regulating overall metabolism.^[3]^ In terms of storage, iron predominantly resides within ferritin.

The prevalence of cardiovascular disease (CVD) shows a significant correlation with both excessive iron accumulation and insufficient iron levels.^[4]^ Among the various manifestations of CVD, acute myocardial infarction (AMI) is the most fatal.^[5]^ There is an ongoing debate regarding the potential role of serum ferritin as a risk factor for coronary heart disease.

In 1981, Zhou et al^[6]^ reported that high levels of stored iron pose a risk factor for the development of cardiac disease, and subsequent studies have demonstrated the strong association between high levels of iron stores and the incidence of AMI.^[7]^ However, some scholars have suggested that elevated iron stores and coronary heart disease may coexist without a direct causative relationship. In a prospective follow-up study in 2001, Ascherio et al^[8]^ reported that individuals who engage in long-term blood donation exhibit notably reduced levels of stored iron and that there is no significant association between blood donation practices and the incidence of coronary heart disease. In a case-control study, Ekblom et al,^[9]^ demonstrated that ferritin does not serve as a risk factor for the onset of AMI. Owing to the controversial results, further investigation of the correlations between these variables is necessary.

Mendelian randomization (MR) is a novel and contemporary scientific research technique that employs genotypes to deduce the connection between phenotype and disease. This research approach relies on the Mendelian principle of random allocation of parental alleles to progeny throughout meiosis. Moreover, MR is recognized as an inherent randomized controlled trial that enables the mitigation of confounding variables and the reversal of causation to a higher degree. By minimizing the influence of confounders and eliminating the possibility of reverse causality, the MR method ensures greater accuracy in evaluating associations between genotype and phenotype, and it is considered a highly reliable approach.^[10]^ Therefore, the causal relationship between ferritin and myocardial infarction (MI) was analyzed by an MR approach in the present study.

## 2. Materials and methods

### 2.1. Study design

**Figure 1.**
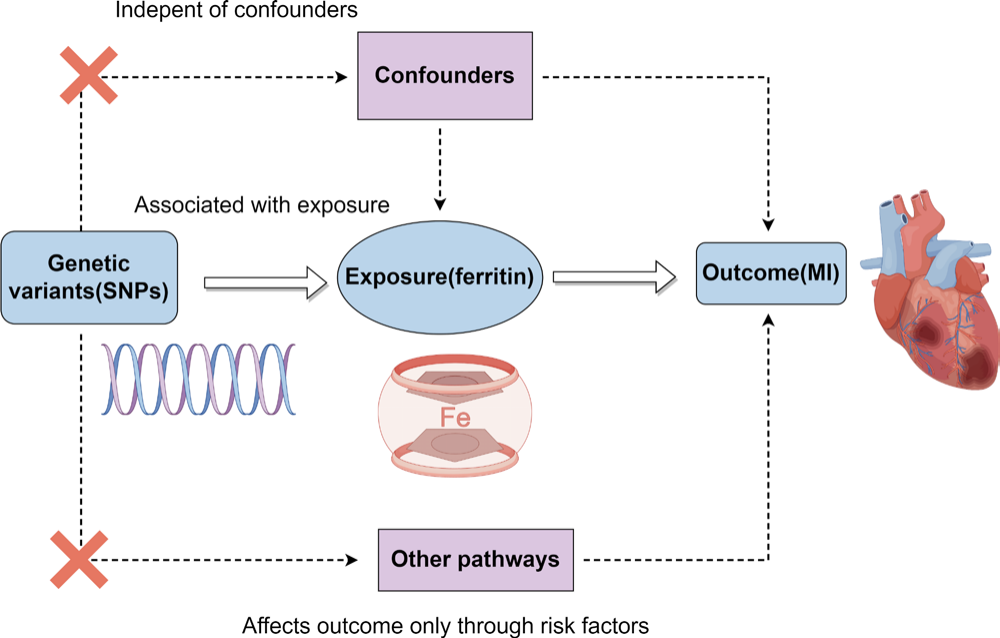
The causal relationship between ferritin and MI was explored by MR. The concepts and 3 core assumptions of the MR analysis are shown in the schematic, which was generated using www.figdraw.com. MI = myocardial infarction, MR = Mendelian randomization.

The present MR study was required to meet 3 essential assumptions, namely, correlation, independence, and exclusion, as follows: the instrumental variable must exhibit a strong correlation with the exposure factor; the instrumental variable must not display any correlation with any confounding factor that is connected to the exposure-result association; and the instrumental variable can solely impact the outcome variable via the exposure factor (Fig. 1). Information was obtained from openly accessible databases (OpenGWAS and Finnish Biobank), which have received informed consent from participants and have been granted ethical approval.

### 2.2. Data source

Ferritin data were obtained from a genome-wide association study (GWAS) dataset (identifier ieu-a-1050) from the IEU Open GWAS database (https://gwas.mrcieu.ac.uk/), and MI data were obtained from 2 different GWAS datasets (identifiers ieu-a-798 and ukb-saige-411.2) from CARDIoGRAMplusC4D (http://www.cardiogramplusc4d.org/) and LeeLab (https://leelab.org/proteomics-and-genomics/). The detailed summary information is presented in Table 1.

**Table 1:** Characteristics of data used in the Mendelian randomization study.

Exposure/Outcomes	GWAS ID	Ethnicity	Total sample size	Source
Ferritin	ieu-a-1050	European	23986	IEU
Myocardial infarction	ieu-a-798	Mixed	171875	CARDIoGRAMplusC4D
Myocardial infarction	ukb-saige-411.2	European	388806	LeeLab

GWAS = genome-wide association study.

### 2.3. Instrumental variable (IV) selection criteria

Independent genetic variants that displayed genome-wide significance (*P* < 5 × 10-8), specifically single nucleotide polymorphisms (SNPs), were selected from the dataset. To ensure the independence of these SNPs, linkage disequilibrium (correlation coefficient r^2^ < 0.001) was eliminated within a genetic distance of kb = 10,000. Following this criterion, the exposed IVs were determined by intersecting the SNPs with the ending SNPs and removing any palindromic SNPs with intermediate gene frequencies. The F value statistic was calculated to evaluate the strength of the IV. If the F value statistic was > 10, then the IV was considered a strong IV; otherwise, it was considered a weak IV.

### 2.4. MR analysis

The association between ferritin and outcomes of MI was investigated using various approaches, namely, inverse variance-weighted (IVW),^[11]^ weighted median,^[12]^ weighted mode,^[13]^ and MR Egger methods.^[14]^. The IVW method was used if Cochrane Q test was *P* < .05, and the IVW fixed model was used if Cochrane Q test was *P* > .05.^[15]^

### 2.5. Sensitivity analysis

To assess the reliability of the MR findings, a sensitivity analysis was conducted. Cochran Q test was utilized to identify discrepancies in the data, with a significance level of *P* < .05 indicating heterogeneity. To detect horizontal pleiotropy and outliers, the MR-Pleiotropy RESidual Sum and Outlier (PRESSO) technique was utilized. Identified outliers were eliminated, and an outlier-corrected MR analysis was conducted to obtain an impartial causal estimation. To evaluate the presence of directional pleiotropy, the MR Egger intercept test was employed. A leave-one-out test was performed to evaluate whether the presence of a SNP significantly affects the MR results. The TwoSampleMR package in R (version 0.5.6) was utilized for the MR analysis, while the MR-PRESSO technique was performed using the MR-PRESSO package in R (version 4.3.2).

## 3. Results

**Figure 2.**
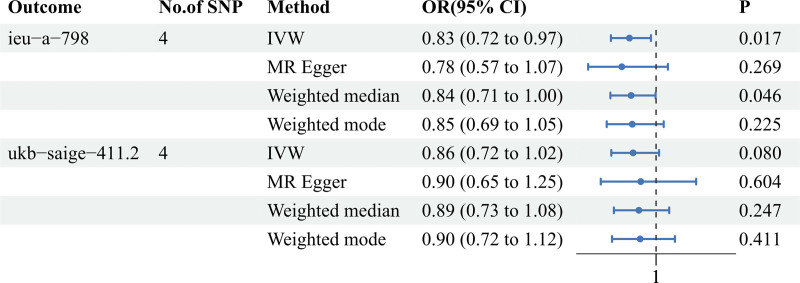
Forest plot of the causal relationship between ferritin and MI. Ferritin may be a protective factor in the incidence of MI. MI = myocardial infarction.

**Figure 3.**
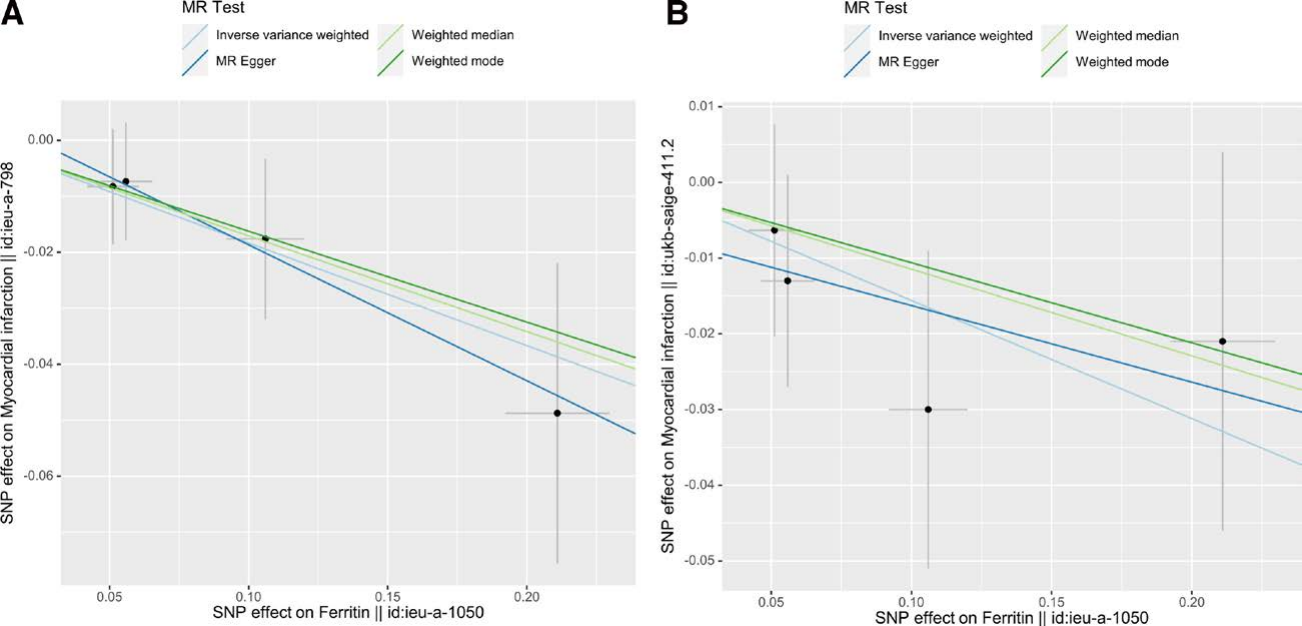
(A) Scatter plot showing the causal relationship between ferritin and MI (Outcome ID: ieu-a-798). (B) Scatter plot showing the causal relationship between ferritin and MI (Outcome ID: ukb-saige-411.2). MI = myocardial infarction.

To strengthen the credibility of the MR study, 2 distinct GWAS datasets related to MI were employed. The F-statistics for all SNPs were >10, indicating the absence of weak IVs. The summary data for MI datasets revealed 4 and 3 SNPs associated with the ferritin dataset. Regarding the causal relationship between ferritin and MI, the first dataset analysis (Outcome ID: ieu-a-798) indicated a statistically significant negative correlation. The second dataset analysis (Outcome ID: ukb-saige-411.2) indicated a negative correlation, but there was no statistical significance. The IVW results showed an odds ratio (OR) of 0.83 [95% confidence interval (CI) = 0.72–0.97; *P* = .017] in the first analysis and an OR of 0.86 (95% CI = 0.72–1.02; *P* = .080) in the second analysis. The weighted median analysis suggested statistical significance in the first dataset, while the remaining 2 MR analysis methods did not show statistical significance. The MR results for ferritin and the 2 MI datasets are presented in Figure 2, and the scatter plots are shown in Figure 3A and B.

**Figure 4.**
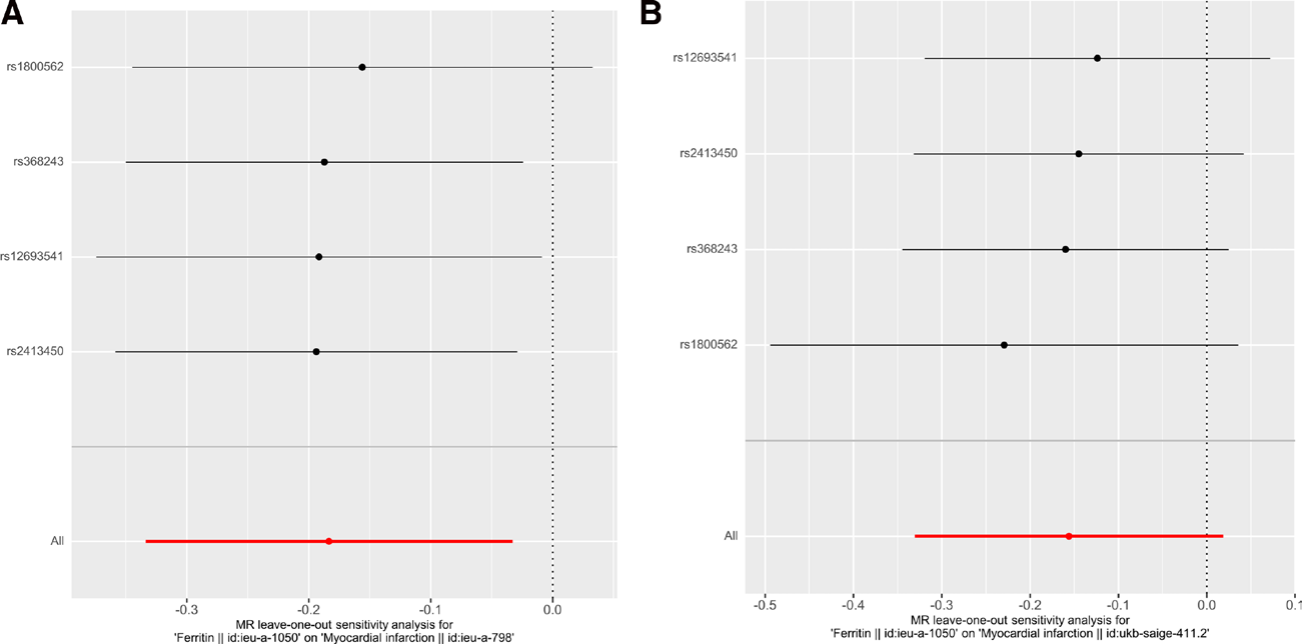
(A) Culling map of SNPs associated with ferritin and MI (Outcome ID: ieu-a-798). (B) Forest map of SNPs associated with ferritin and MI (Outcome ID: ukb-saige-411.2). MI = myocardial infarction, SNP = single nucleotide polymorphism.

To evaluate heterogeneity in the data, Cochrane Q test (IVW and MR Egger methods) was utilized. There was no heterogeneity in either group, as indicated by *P* values of .970 and .862 for the IVW method and *P* values of .967 and .742 for the MR Egger method. The MR Egger intercept method, which was used to determine directionality pleiotropy, yielded *P* values of .714 and .73, suggesting that MI was not influenced by IVs through pathways other than ferritin. MR-STRO was employed to confirm the absence of horizontal pleiotropy and outliers in both datasets (*P* = .966 and *P* = .812). Additionally, the leave-one-out test demonstrated stability (Fig. 4A and B).

**Figure 5.**
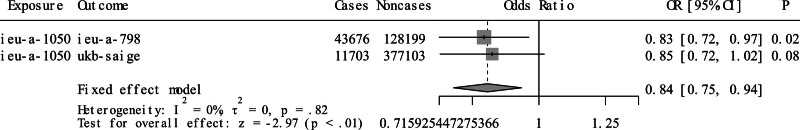
Forest plot for the meta-analysis of the 2 MI datasets. MI = myocardial infarction.

To obtain more robust evidence, a meta-analysis was conducted using the 2 datasets. The heterogeneity was assessed using the I^2^ test, which indicated the absence of heterogeneity between the 2 datasets (*P* = .82 and I^2^ = 0% < 50%). Consequently, a fixed-effects model was employed, which demonstrated a negative association between ferritin and MI (OR = 0.84 with a 95% CI = 0.75–0.94, *P* < .01) (Fig. 5).

## 4. Discussion

The present study employed the MR method to systematically evaluate the causal connection between the genetic susceptibility to ferritin and the likelihood of developing MI. The genetic prediction analysis indicated a significant association between ferritin levels and a decreased risk of MI. To verify the MR approach, 2 MI datasets were subjected to a comprehensive meta-analysis, which corroborated the initial findings, thereby validating the reliability of the research outcomes.

The serum iron levels in the human body are highly unstable and susceptible to various factors, and they do not accurately reflect the body iron storage levels.^[16]^ Serum ferritin, a protein containing iron ions, serves as the primary form of iron storage in the body and maintains a stable level. Serum ferritin is commonly used as an objective indicator to assess the body iron storage content.^[17]^

Previous studies have indicated an increased risk of AMI associated with ferritin. For example, individuals with serum ferritin levels >200 μg/L have a 2.2-fold higher risk of experiencing AMI compared to those with lower levels. For every increase of 100 μg/L in serum ferritin, the risk of AMI increases by 0.2 times.^[7]^ In MI models, a decrease in the level of ferritin heavy chain 1 (FTH1) at the infarct site leads to elevated levels of free iron and oxidative stress, which promotes iron-mediated cell death, resulting in myocardial cell death and the occurrence of MI.^[18]^ In addition, GPX4 protein levels are reduced in MI tissues compared to normal tissues, leading to iron death and ultimately to the development of AMI.^[19]^

Although most studies have suggested that ferritin is a causative factor of atherosclerosis and MI, some studies have proposed that iron overload does not promote atherosclerosis but instead may reduce atherosclerosis.^[20]^ Habib et al reported that the hemorrhage area of iron-rich plaques show lipid retention, inflammation, and reduction of reactive oxygen species (ROS).^[21]^ Moreover, oxidative stress induced by iron overload induces cellular damage, but ferritin protects cells from redox activity.^[22]^

Some studies have identified the potential for ferritin or ferritin-related pathways to improve MI. Han et al reported that ferritin nanoparticles (IONPs) significantly enhance the expression of connexin 43 (Cx43), an intercellular gap junction protein, in cardiomyocytes, which is essential for generating an improved therapeutic potential when co-cultured with mesenchymal stem cells (MSCs).^[23]^ These measures for treating MI may be related to the properties of ferritin. By employing lipid spherical proton magnetic resonance contrast agents, such as HA-NWs, in patients with congestive heart failure, Nasr et al^[24]^ detected atherosclerotic plaques noninvasively by magnetic resonance imaging, highlighting the potential value of ferrite nanoparticles in the treatment of CVDs, including MI. These previous studies suggest the potential of ferritin in improving MI.

Given the limited number of studies on ferritin and MI, it is essential to conduct large-scale prospective studies and gather further evidence on the underlying mechanisms. Although the present MR study provided valuable insights, there were several limitations. The study population primarily consisted of individuals of European ancestry, thus restricting the generalizability of the present findings to other countries or ethnicities. Additionally, the lack of detailed clinical information prevented subgroup analysis to determine the specific causal relationship.

## 5. Conclusions

In summary, the MR study provided insights into the causal relationship at the genetic level and demonstrated a negative correlation between ferritin and the risk of MI, thereby offering new perspectives for future treatments in AMI.

## Acknowledgments

We express gratitude to OpenGWAS, CARDIoGRAMplusC4D, and the LeeLab database for providing the datasets.

## Author contributions

**Conceptualization:** Jianwei Zhou.

**Data curation:** Jianwei Zhou.

**Formal analysis:** Jianwei Zhou.

**Funding acquisition:** Jianwei Zhou.

**Investigation:** Jianwei Zhou.

**Methodology:** Jianwei Zhou.

**Project administration:** Jianwei Zhou.

**Resources:** Jianwei Zhou.

**Software:** Jianwei Zhou.

**Supervision:** Jianwei Zhou.

**Validation:** Jianwei Zhou.

**Visualization:** Jianwei Zhou.

**Writing – original draft:** Jianwei Zhou.

**Writing – review & editing:** Jianwei Zhou.
